# Barriers and Willingness to Continue Using Telehealth Services Beyond the COVID-19 Pandemic from the Perspectives of Oral and Maxillofacial Surgeons in Australia: A Mixed-Method Study

**DOI:** 10.3390/healthcare12202086

**Published:** 2024-10-19

**Authors:** Chipampe Masongo, Judith Daire, Mohamed Estai, Dieter Gebauer, Leon Smith, HuiJun Chih

**Affiliations:** 1 Curtin School of Population Health, Curtin University, Bentley, WA 6102, Australia; 2 School of Human Sciences, The University of Western Australia, Crawley, WA 6009, Australia; 3 Department of Oral and Maxillofacial Surgery, Royal Perth Hospital, Perth, WA 6000, Australia

**Keywords:** telemedicine, telehealth, COVID-19, specialist care access, barriers, oral and maxillofacial surgery, remote consultation

## Abstract

Background: Patient demand for oral and maxillofacial telehealth services increased during COVID-19. To explore the potential for their continued use post-COVID-19, an assessment was conducted by examining the association between the clinical and socio-demographic characteristics of consultants and the perceived facilitators and barriers influencing their future intent. Methods: Practicing oral and maxillofacial consultants were recruited through purposive and snowball sampling methods. Data were collected through surveys and key informant interviews. Chi-square tests were used to determine whether consultants’ clinical and socio-demographic characteristics and perceptions were associated with a willingness to use telehealth in the future. Coded interview transcripts were analyzed thematically to identify the main themes influencing their willingness. Results: Among the 42 respondents, 82% expressed a willingness to continue using telehealth services with the majority having at least 2 to 3 years (*p* = 0.028) of experience utilizing these services. The four main themes impacting consultants willingness include the accessibility of oral and maxillofacial healthcare, challenges addressing patient needs, the uncertainty of diagnostic accuracy and effectiveness as a post-operation observation tool. Conclusion: Most oral and maxillofacial consultants favored the routine use of telehealth services beyond COVID-19. Concern for patients’ needs was a key determinant of their continued use. Co-designing strategies to eliminate barriers and unmet needs for consultants and patients may improve oral and maxillofacial telehealth uptake.

## 1. Introduction

In March 2020, the World Health Organization declared COVID-19 a pandemic, elevating global efforts against its transmission [1]. This declaration prompted the swift implementation of infection control measures in many countries, including Australia, to protect patients and healthcare professionals [2]. Over three years, COVID-19 profoundly impacted healthcare systems globally [3]. While acknowledging the remaining uncertainties posed by the potential evolution of SARS-CoV-2, recommendations for long-term management marked the transition from a global emergency to a sustained care focus [1]. Government-led initiatives prioritizing quality care aided the integration of telehealth into healthcare delivery.

Telehealth facilitates healthcare delivery through electronic communication for consultations, diagnosis, referrals, and education [4]. Despite initial underutilization in hands-on specialties such as oral and maxillofacial surgery, its usage soared during COVID-19, demonstrating its importance during emergencies and potential for long-term integration into clinical practice [5,6]. This heightened the demand for telehealth use in specialist fields which intensified challenges, in establishing diagnostic accuracy [6]. Patients having more responsibility during the examination process, also raised ethical and legal concerns as they increased the potential for misdiagnosis or delayed treatment [7]. Additional challenges come from technology issues such as unreliable networks, low digital literacy, a lack of proper infrastructure, and potential threats to privacy and consent given the vast amount of patient data that is gathered [8].

Whilst telehealth in oral and maxillofacial healthcare is projected to expand, it is unlikely to fully replace in-person consultations, as indicated by a COVID-19 study involving 20 surgeons [5,6]. However, a comprehensive evaluation of telehealth’s benefits and drawbacks post-pandemic is lacking. Assessing consultants’ willingness to continue telehealth use is crucial, particularly with growing patient demand [5] and the need to provide equitable access to healthcare for patients experiencing access inequalities. Identifying system deficiencies by applying implementation frameworks and models can help overcome barriers and maximize benefits for patients and providers. The Technology Acceptance Model and Theoretical Domain Framework provide valuable insights into the drivers of, barriers to, and predictors of telehealth adoption. The TDF provides a comprehensive framework for understanding behavioral factors in domains like social/professional identity, environmental context and resources, and one’s beliefs about their capabilities [9]. The TAM identifies technology-specific factors that predict adoption based on perceived usefulness and ease of use, providing insight into people’s intentions to use technology [10]. The nature of telehealth in specialized fields prompts this study to incorporate the TDF to identify factors that influence and explain the determinants of oral and maxillofacial surgery telehealth adoption, allowing for a deeper understanding of the challenges and facilitators involved. This study assessed the link between oral and maxillofacial consultants’ characteristics, perceptions, and willingness to sustain telehealth services. Additionally, it delved into consultants’ experiences to identify perceived barriers and unmet needs affecting their ongoing use of telehealth in oral and maxillofacial surgery.

## 2. Materials and Methods

### 2.1. Study Design and Participants

This study utilized an explanatory sequential mixed-method design, incorporating both quantitative and qualitative approaches informed by phenomenology [11]. It aimed to supplement quantitative data with insights into consultants’ experiences and factors affecting their telehealth adoption. Considering our study purpose, the target study population was Australian consultants, and our sampling frames included the membership directory of the Australian and New Zealand Association of Oral and Maxillofacial Surgeons (ANZAOMS) and the research team’s networks. Practicing oral and maxillofacial consultants were recruited through purposive sampling from the ANZAOMS directory and snowball sampling from the networks. These approaches were particularly effective for reaching consultants; however, there are biases associated with both sampling methods, including selection bias based on predefined characteristics and homogeneity, with participants only referring individuals with similar backgrounds [12]. Survey links were distributed to all members of ANZAOMS and their networks in September 2022, which had an estimated cohort of 90 consultants. This membership number represents a substantial portion of the target population as reported by the Australian Health Practitioner Regulation Agency, which listed 176 registered oral and maxillofacial surgeons during the reporting period from 01 July 2022 to 30 September 2022 [13]. Respondents were offered the opportunity to participate in follow-up interviews at the end of the survey. Ethics approval for this study was granted by the Curtin Human Research Ethics Committee (approval number: HRE2022-0317-02).

### 2.2. Instrument

Quantitative and qualitative data were collected sequentially through an online survey and key informant interviews. A 29-question online Qualtrics [14] survey adapted from previous surveys on telehealth utilization during COVID-19 [15,16,17] examined consultants’ perceptions of oral and maxillofacial telehealth. Two practicing consultants piloted the survey to ensure clarity and validity. Their reviews resulted in minor modifications, including text entry instead of option selection for demographic questions. Follow-up interviews aimed to confirm and expand upon survey findings using an interview guide (Table 1) developed from survey responses. Pilot interviews with consultants (who also reviewed the survey) ensured a thorough exploration of barriers and unmet needs in oral and maxillofacial telehealth implementation, complementing survey data.

### 2.3. Data Collection

Demographic data, including age, service type, and state/territory, were collected online via Qualtrics [14]. Information was collected on perceived advantages (i), disadvantages (ii), the necessity of telehealth (iii), experiences with telehealth (iv), and desire to utilize telehealth in the future (v). The first author conducted one-on-one online interviews with five consultants, with the second author supervising three sessions. The Theoretical Domains Framework (TDF) of behavior change guided the study’s exploration of relevant factors influencing consultants’ intentions to utilize telehealth [9]. Data saturation was determined by the repetition of statements and the alignment of qualitative findings with the preceding quantitative data. Audio recordings were made with participants’ consent, and interviews lasted between 12 and 15 min.

### 2.4. Data Analysis

Qualtrics survey responses were exported to CSV files, checked for errors, and analyzed using statistical software (SPSS version 28.01.1, New York, NY, USA) [18]. Demographic characteristics were described using quantities and proportions for categorical variables and means with standard deviations for normally distributed variables and medians with interquartile ranges for skewed variables. Chi-squared tests (or Fisher’s exact tests when assumptions were violated) were employed to evaluate associations between consultants’ characteristics and clinical perceptions with their willingness to sustain telehealth beyond COVID-19, with statistical significance set at *p* < 0.05. Recordings were transcribed and color coded based on participant characteristics. Inductive and deductive approaches were used to analyze themes [19]. Initial codes were developed and documented in a pre-set codebook through a deductive approach, based on interview guides and research questions [20]. An inductive coding method was used to reveal new themes and patterns from the data. NVivo 12 software [21] was used to code the transcripts, identify recurring patterns, and develop themes based on the repeated statements. The themes were reviewed multiple times to ensure clarity, relevance, and comprehension. A comparison of qualitative themes with quantitative results was conducted to enhance the overall rigor of the study and ensure consistency. An audit trail ensured reliability and reporting adhered to the Consolidated Criteria for Reporting Qualitative Research (COREQ) guidelines [22].

## 3. Results

A total of 42 consultants of the 90 invited consultants participated in the study, yielding a response rate of 46.6%. This sample size was deemed sufficient for this exploratory study because the total number of registered oral and maxillofacial surgery consultants in Australia was small (n = 176) and consistent with response rates in prior telehealth studies among specialists [13,23]. The analysis included four partially completed surveys of the 42 respondents. Five consultants further participated in subsequent interviews as key informants.

### 3.1. Demographic and Clinical Background of Respondents

Among the 42 consultants, the average number of years of experience was 20 years (SD: 13.7), with 72% being dual-qualified in medicine and dentistry and 69% practicing for over 11 years. The majority were male (86%) and fell within two main age categories: 45 to 54 (26%) and 55 to 64 (26%) (Table 2). Three-quarters of the consultants’ primary practice locations were in Victoria, followed by New South Wales, Queensland, South Australia, Western Australia, and Tasmania. None practiced in any of the Australian territories.

This mixed-method analysis intertwines the survey data and subsequent interviews to explain the survey findings and explore factors impacting consultants’ telehealth adoption. The following four key themes emerged: (i) the accessibility of oral and maxillofacial healthcare, (ii) challenges addressing patient needs, (iii) the uncertainty of diagnostic accuracy, and (iv) post-operation observation tools, as detailed in Table 3 alongside the associated factors, consultant quotes, and the relevant TDF domains.

### 3.2. Accessibility of Oral and Maxillofacial Healthcare

**Figure 1:**
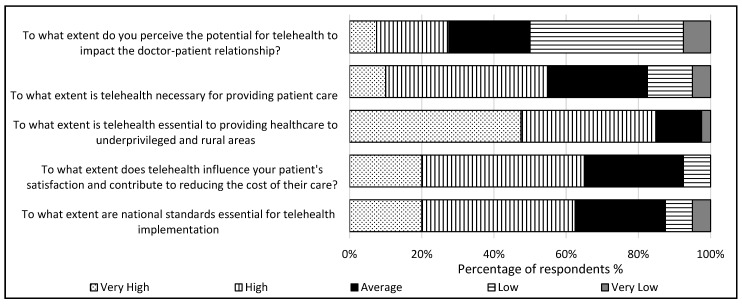
Consultants’ perceptions of telehealth utilization (n = 38).

In regions with fewer consultants available, telehealth has mitigated access challenges and enhanced patient access, satisfaction, convenience, and acceptance. The survey results revealed consultants’ considerable appreciation of factors aligning with patient needs. Specifically, 65% agreed that reducing patient costs and enhancing patient satisfaction constitute notable advantages of telehealth (Figure 1). Moreover, 55% perceive telehealth as either highly or very highly necessary for providing oral and maxillofacial healthcare. This sentiment was reinforced by the fact that 85% of the respondents acknowledged that telehealth is essential in delivering quality healthcare to marginalized groups. Additionally, 62.5% highlighted the significance of national standards for oral and maxillofacial telehealth services.

**Figure 2:**
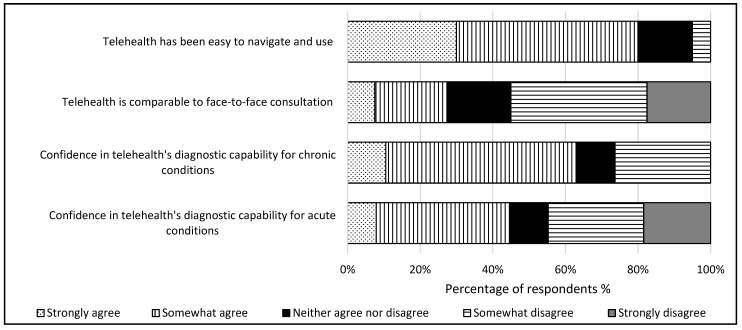
Consultants’ experiences with telehealth utilization (n = 38).

The consultants’ perceptions of telehealth use during COVID-19 revealed that the perceived ease of navigation and their willingness to continue using telehealth were significantly related (*p* = 0.041), in which the consultants agreeing that telehealth was easy to navigate was associated with them expressing a desire to continue its use (Figure 2).

The interviews revealed that most consultants perceive telehealth as beneficial for enhancing access to oral and maxillofacial healthcare, especially in rural and remote areas. The results identified that the consultants predominantly work in both private and public settings, and the interviews found telehealth is beneficial for the consultants in reaching patients at their primary location and expanding access to patients in areas with limited specialist availability in other states and/or territories (Table 3). The consultants emphasized that telehealth enhances the convenience of care for rural and remote patients, improving treatment efficiency and recovery. They stressed the importance of sufficient technical infrastructure and support for telehealth services, particularly in these settings. An emphasis was placed on the necessity of MBS-funded telehealth services and ample patient resources in remote areas (Table 3).

### 3.3. Challenges to Addressing Patient Needs

This theme delves into how patients’ attitudes toward telehealth influence consultants’ perceptions and willingness to continue its use, noting challenges in patient interactions impacting their desire to continue. The subthemes identified were (2.1) the doctor–patient relationship and (2.2) devalued consultation method (Table 3). The consultants’ views on whether telehealth disrupts doctor–patient relationships were divided, with half (50%) indicating low disruption (Figure 2). A small portion (22.5%) perceived telehealth in oral and maxillofacial healthcare as a source of malpractice. However, the majority (72.5%) did not see telehealth as a significant hindrance to patient care delivery.

#### 3.3.1. Doctor–Patient Relationship

In their diverse experiences with telehealth, the consultants highlighted the challenge of establishing a robust patient–doctor relationship. Acknowledging the intricacies of their profession, the consultants emphasized the importance of patient trust and comprehension of treatment implications. Without physical cues and signs present in face-to-face consultations, it was found to be challenging to predict patient expectations (Table 3).

#### 3.3.2. Devalued Consultation Method

The results showed that using telehealth beyond COVID-19 is inversely associated with the consultants’ years of prior usage (*p* = 0.028). Of those who would continue using telehealth, 62% have at least 2–3 years’ experience, with the remainder distributed between those with 0–1 and >3 years of experience. A significant association emerges between the consultants’ views on telehealth’s equivalence to face-to-face consultations and their inclination towards its continued use. Notably, all respondents expressing reluctance towards telehealth strongly opposed its comparability to face-to-face consultations (*p* = 0.004). Moreover, consultants facing disruptions in doctor–patient communication via telehealth show less eagerness to use telehealth in the future (*p* = 0.001). To explore factors behind the survey results, the interviewees were asked about telehealth’s disadvantages, comparisons with face-to-face consultations, and widespread adoption challenges. The consultants reflected on their experiences of telehealth use in practice, discovering that patients did not perceive the same value in telehealth consultations as they did in face-to-face consultations (Table 3). The concept of a devalued consultation method became clear during COVID-19, with the consultants noting that patients did not appear to treat telehealth consultations as real consultations, which for some led to perceptions of a worse delivery of oral and maxillofacial healthcare (Table 3).

### 3.4. Uncertainty of Diagnostic Accuracy

The consultants’ assurance in utilizing telehealth to assess chronic (*p* = 0.009) or acute (*p* = 0.024) conditions significantly influenced their willingness to use it. Most consultants, 64%, express confidence in telehealth’s diagnostic proficiency for chronic conditions, while 45% report satisfaction with its diagnostic capabilities for acute conditions (Figure 2). The consultants voiced concerns regarding the accuracy of patient examinations through telehealth, emphasizing its suitability depending on the patient’s condition (Table 3). They noted a prevalence of telephone consultations over video consultations in oral and maxillofacial surgery. While technological advancements aid in examinations, the intricacies of the oral cavity pose challenges for accurate diagnosis without physical examination. Consequently, consultants often opt for in-person assessments or conduct additional tests before treatment (Table 3).

### 3.5. Post-Operation Observation Tools

The consultants found telehealth beneficial for post-operative follow-ups, particularly in managing and reviewing patients after oral and maxillofacial treatment (Table 3). They employ a sequential healthcare delivery model, offering face-to-face initial consultations and telehealth follow-ups after treatment. The consultants agreed that this approach ensures they possess a comprehensive clinical history of their patients for effective telehealth consultations (Table 3).

## 4. Discussion

Oral health professionals show growing interest in telehealth, particularly for remote patients [16,24]. This study observed that there is a willingness among Australian oral and maxillofacial consultants to continue using telehealth services in the future with 82% of the consultants from this study expressing their willingness to use it routinely. This finding complements previous research findings, stating telehealth is crucial to providing healthcare to vulnerable patients [6,25]. However, our study found that as their experience with telehealth increases, the consultants are less willing to continue its use in the future. Whilst this finding contradicts that of other research, interview data from the consultants revealed that patient attitudes strongly influenced their decision to discontinue telehealth use. It was revealed that these barriers and unmet needs stem from challenges encountered in addressing patients’ needs, similar to findings from a study in Florida [16]. Considering these findings, patients’ needs must be considered and met, since they represent the primary motivating factor for consultants continued use of telehealth services. When consultants can effectively address distant patients’ needs via telehealth, its usage rises [24]. Studies have highlighted the benefits of telehealth in enhancing accessibility and saving time in clinical practice [25]. Time-efficient delivery can be equated with the need for technical infrastructure and funding for telehealth delivery and accessibility. The consultants in this study expressed a strong willingness to continue using telehealth if these requirements are met, citing factors like its ease of navigation, suitability for assessing various conditions, and seamless patient communication as supporting factors. The TDF domains provided insight into drivers and obstacles influencing the consultants’ telehealth adoption. These behavioral and psychological factors reveal the consultants’ inclinations, which are central in shaping their readiness to embrace telehealth in future practice. It is possible to guide, enhance, and incorporate telehealth policies and strategies more effectively without compromising quality by providing funding for technical infrastructure in low-served areas, diagnostic support tools, and targeted training for consultants, which address the domains of environmental context and resources, beliefs and capabilities, knowledge, goals, and memory, attention, and decision processes.

Without the ability to perform a physical examination, oral and maxillofacial surgeons face challenges when assessing patients via telehealth [6]. Consultants express low confidence in telehealth’s diagnostic capabilities, citing the unpredictability of patient-reported symptoms (60%). Their reluctance to sustain telehealth also stems from challenges in establishing doctor–patient relationships, as without direct face-to-face interactions, vital nonverbal information can be overlooked [7]. This is further exacerbated by practical technological difficulties, which include poor connectivity, inexperience with digital platforms, software compatibility issues, and access issues, all of which hinder communication and compromise healthcare quality [6,7]. In the absence of a comprehensive response to these relational challenges, consultants remain hesitant to incorporate telehealth into their regular practice. Providing comprehensive technical support to both patients and consultants is therefore important. Education and instructional support for patients in the form of tutorials and technical assistance can ensure that consultation quality is not adversely affected by technical challenges. This study affirmed that the TDF domains reflect aspects of telehealth use that influence consultants’ decisions. The implementation of telehealth policies and strategies that address these domains, such as funding for technical infrastructure in low-served areas, diagnostic support tools using artificial intelligence, and targeted training for consultants, will allow telehealth to be incorporated more effectively without compromising quality.

Most consultants perceived telehealth to have a low malpractice risk in surveys. However, privacy concerns persist, as evidenced in interviews with consultants, who indicated they preferred in-person consent. Integrating telehealth effectively in specialist fields requires enhancing virtual communication and safeguarding patient privacy. Reviewing standardized procedures and guidelines for oral and maxillofacial telehealth that incorporate informed consent procedures similar to those found in traditional settings may alleviate ethical concerns. The present findings are consistent with the following recommendations which are currently used and remain valuable guidelines for telehealth integration. The first is postoperative follow-up, which increases with adequate resources, thus meeting demand, improving resource allocation, and maximizing patient care. The second approach involves conducting all consultations via telehealth with a thorough assessment of the patient’s condition. Consultants have deemed this option effective, but it depends on carefully evaluating the patient’s condition and resource availability. The success of this approach is contingent upon the patient’s condition and remains untested. A practical application of the TAM supports these recommendations, emphasizing its perceived usefulness and ease of use as predictors of continued telehealth use [10]. Following these concepts, consultants consider telehealth useful in increasing access to care compared to traditional face-to-face consultations. The accessibility of telehealth has motivated oral health professionals to incorporate it into their practices, especially when it is used to meet their patients’ long-distance needs [24]. Despite its usefulness, many consultants remain hesitant to use telehealth as they cite barriers to its usability, including maintaining doctor–patient relationships, the perception of its value by patients, and limitations in its diagnostic capabilities. Consequently, consultants’ perceptions of its ease of use are reflected in the reduction in its actual use over time, as evidenced by our findings. As telehealth is perceived as useful but its ease of use is poor, improving integration should emphasize enhancing its usability without compromising the key benefits perceived as useful.

A successful oral and maxillofacial telehealth service must prioritize patients’ and clinicians’ needs while also addressing organizational factors for safety and reliability [25]. Concerns about telehealth’s diagnostic capabilities from consultants highlight the importance of adhering to remote treatment standards and guidelines, enhancing telehealth’s viability [26,27]. There is a critical need to ensure that patients are knowledgeable about telehealth consultations to keep their value comparable to in-person consultations. Promoting and communicating telehealth programs is crucial for achieving this goal [27].

### Strengths and Limitations of the Study

This study examines multiple factors influencing oral and maxillofacial consultants’ readiness to sustain telehealth services across Australia using a mixed-method approach incorporating the TDF framework, validated surveys, interviews, and scientific analyses to illustrate the facilitators of telehealth, barriers to telehealth, unmet needs, and recommendations to enhance its integration. Despite our collaboration with ANZAOMS, female consultants and those from outside Victoria and New South Wales are minimally represented, limiting the generalizability of our findings. The lack of diversity in this sample could skew the results, making generalization difficult. A more diverse sample of genders and regions should be included in future research to gain a more precise understanding of telehealth’s effectiveness across demographics; for example, the insights of a more diverse group of consultants could lead to improvements and broader service delivery. The purposive and snowball sampling strategies effectively reached relevant consultants; however, they may have limited the diversity of perspectives. Therefore, the conclusions from this study may mainly reflect the perspectives of the consultants included, rather than broader demographics. To address these concerns, future research could combine purposive sampling with random sampling techniques and diversify recruitment channels in snowball sampling to increase representativeness. Although this study does not consider patient perspectives, incorporating them into future studies could improve telehealth implementation. Engaging patients in discussions with consultants could help develop strategies to address barriers and unmet needs. The patient perspective is crucial to understanding the strengths and weaknesses of telehealth services. This helps identify areas for improvement, ensuring that telehealth meets both the needs of patients and consultants.

## 5. Conclusions

Consultants view telehealth positively and are willing to continue its routine use post-COVID-19, particularly for post-operative follow-ups. Their prior telehealth experience influences their readiness, confidence in its accuracy, efficiency, and integration into clinical practice. Addressing patient needs is crucial for sustaining telehealth use. Collaborative strategies to address barriers and unmet needs for consultants and patients can enhance oral and maxillofacial telehealth adoption.

## Figures and Tables

**Table 1:** Interview guide.

Question
What do you think are the advantages and disadvantages of using telehealth for a consultation?
Do you prefer using telehealth or face-to-face consultations? Could you tell me why this is?
In your current role, could you tell me if there are reasons some patients are deemed unsuitable for telehealth consults?
Adding onto this, what do you think were the main challenges to the wider adoption of telehealth before the COVID-19 pandemic?
What do you think the major barriers to integrating telehealth in clinical practice are now?
Do you think these barriers will remain after the COVID-19 pandemic?

**Table 2:** Description of clinical and socio-demographic characteristics of consultants (n = 42).

Characteristic	Frequency (n, %)
Age (Years)	
20–34	7 (17)
35–44	7 (17)
45–54	11 (26)
55–64	11 (26)
≥65	6 (14)
Gender	
Male	36 (86)
Female	6 (14)
Work settings	
Public Hospital	1 (2.4)
Private Practice	11 (26.2)
Both	29 (69)
Other	1 (2.4)
Years of experience	
1 to 10 yrs	13 (31)
11 to 30 yrs	21 (50)
>30	8 (19)
Location of primary job (State/Territory)	
NSW	9 (21.4)
VIC	14 (33.3)
QLD	8 (19)
TAS	1 (2.4)
SA	5 (11.9)
WA	5 (11.9)
Years using telehealth in practice	
0–1 years	5 (12)
2–3 years	26 (62)
>3 years	11 (26)

**Table 3:** Consultants’ willingness to use telehealth, their quotes, and the TDF domains reflecting their desires.

Factors Associated with Consultants’ Willingness to Continue Telehealth Use and Their Respective Quotes (Including Gender and Years of Experience)	TDF Domain
1. Accessibility of oral and maxillofacial healthcare	
“Some convenience factors that are advantageous is the distance aspect, not having to travel for an appointment” (male, 22 years of experience). “It’s a benefit and plays a good role for patients that are from a great distance. They appreciate it” (male, 14 years of experience). “I commonly have patients coming from places that are a 5-h drive away, additionally from Western Australia, Southern Australia and the Northern Territory, so it is often ideal talking to them over a telehealth” (male, 55 years’ experience) “Barriers in the legislation and infrastructure put people (consultants) off …….. if they withdraw telehealth off the Medicare schedule, it will disappear, which would be unfortunate it would be a terrible thing because it’s something doctors have been pushing for a long time to be able to do this” (male, 7 years of experience) “But for many of us that have country practice or rural practices where we need to look after patients from a distance, it’s vital, and I’d assume that will continue.” (male, 14 years of experience)	Knowledge Beliefs and capabilities Reinforcement Goal Environmental context and resources Memory, attention, and decision process
2. Challenges to Addressing Patient Needs.	
2.1 Doctor–patient relationship	
“There is no doubt that when you are interacting directly with the person, and you get a lot from that, then you can get over telehealth” (male, 55 years of experience) “It breaks down the patient-doctor barrier. I don’t feel the connection you make between you and your patient is strong. I don’t feel the patient has an engagement psychologically; I think it is a cursory, superficial engagement.” (male, 22 years of experience) “Obtaining consent from them and explaining things to them is probably more ideal in person” (male, 14 years of experience) “The sort of non-verbal cues that people get from face-to-face interactions are hard to get over telehealth” (male, 22 years of experience)	Beliefs and consequences Emotions Intention Environmental context and resources
2.2 Devalued Consultation Method	
“… there is no concept of the value. People refuse to pay because they don’t consider that it’s a full consultation” (female, 35 years of experience) “…people don’t answer their phones when you call them at the appointment time” (male, 22 years of experience) “People will fiddle around with downloading, the ease of which it is conducted is more difficult” (female, 35 years of experience) “You provide a very valuable service during very trying circumstances, and people are just doctor shopping going around over telehealth” (male, 22 years of experience)	Beliefs and consequences Emotions Intention Environmental context and resources
3. Uncertainty of Diagnostic Accuracy	
“Clinical problems relating to facial pain, historical matters, medicine, surgical pathologies, and logistic difficulties telehealth is good for that” (male, 55 years of experience) “For patients with complex treatment management and plans, patients with comorbidities and complex medical history, it is better to have them come in” (male, 14 years of experience) “An obvious disadvantage, particularly in oral and maxillofacial, is examination. The oral cavity is very challenging over telehealth” (male, 7 years of experience) “When you have never seen a patient before, telehealth becomes particularly difficult” (male, 14 years of experience) “Examining someone is a requisite” (male, 22 years of experience)	Social/professional role and identity Optimism Beliefs about consequences Environmental context and resources
4. Post-operation Observation Tool	
“It helps with …………… post-operative review appointment” (male, 22 years of experience) “I don’t see most patients post-operatively; I use telehealth for postop consultation” (female, 35 years of experience) “Where we have multiple follow-up appointments……… It helps with recall and ensuring they are fine whilst providing formal information back to the patient. Doing that over telehealth is ideal” (male, 14 years of experience) “It is best for review, patients whom you’ve seen physically once, so you know what their background is then it saves everybody a lot of time having a chat with them by telehealth” (male, 55 years of experience) “It’s highly convenient for post-operative contact and pathology review for minor procedures” (male, 7 years of experience)	Skills Intention Goal Behavioural regulation Memory, attention, and decision process

## Data Availability

The original contributions presented in the study are included in the article/Supplementary Material; further inquiries can be directed to the corresponding author.

## Supplementary Materials

The following supporting information can be downloaded at: https://www.mdpi.com/article/10.3390/healthcare12202086/s1, Table S1: Association between consultants’ clinical and socio-demographic characteristics and willingness to continue using telehealth (n = 38); Table S2: The association between consultant perceptions of telehealth use in practice and desire to continue using telehealth (n = 38).
