# Supplementary material for: Barriers and Willingness to Continue Using Telehealth Services Beyond the COVID-19 Pandemic from the Perspectives of Oral and Maxillofacial Surgeons in Australia: A Mixed-Method Study
Source: Healthcare (Basel). 2024 Oct 19;12(20):2086. doi: 10.3390/healthcare12202086 (PMC11507098; doi:10.3390/healthcare12202086)
Supplement: Supplementary file 1 [file healthcare-12-02086-s001.zip › healthcare-3239218-supplementary.pdf]

Table S1: Association between consultants' clinical and socio-demographic characteristics and willingness to continue using telehealth (n=38)

	**Willingness to continue the use of telehealth**			
**Clinical and socio-demographic characteristics**	Yes (%)	No (%)	Unsure (%)	*p-value*
Years of Experience				
1 to 10	10 (32.3)	0	0	0.287
11 to 30	14(45.2)	3(100)	3(75)	
30+	7(22.6)	0	1(25)	
Location of the main job				
NSW	6(19.4)	0	1(25)	0.364
VIC	12(38.7)	1(33.3)	1(25)	
QLD	5(16.1)	0	1(25)	
TAS	1(3.2)	0	0	
SA	5(16.1)	0	0	
WA	2(6.5)	2(66.7)	1(25)	
Job setting(s)				
Public hospital	1(3.2)	0	2(50)	0.841
Private hospital	8(25.9)	0	2(50)	
Both	21(67.7)	3(100)	0	

Other	1(3.2)	0	0	
Prior telehealth use				
0-1 years	4(12.9)	0	1(25)	*0.028
2-3 years	22(71)	1(33.3)	0	
>3 years	5(16.1)	2(66.7)	3(75)	
Frequency of telehealth use before COVID-19				
Frequently (>1 monthly or >12 yearly)	7(22.6)	0	0	0.491
Occasionally (1- 12 times a year)	11(35.5)	2(66.7)	3(75)	
Never	13(41.9)	1(33.3)	1(25)	

The test applied: Fisher's exact tests, **P* <0.05 considered statistically significant *N*

Table S2 The association between consultant perceptions of telehealth use in practice and desire to continue using telehealth (n=38)

	Willingness to continue the use of telehealth			
**Consultant perception**	Yes (%)	No (%)	Unsure (%)	*p-value*
Telehealth has been easy to navigate and use				
Somewhat disagree	0	1(33.3)	0	*0.041
Neither agree nor disagree	3(9.7)	1(33.3)	2(50)	
Somewhat agree	17(54.8)	1(33.3)	2(50)	
Strongly agree	11(35.5)	0	0	
Telehealth is comparable to face-to-face consultation				
Strongly disagree	2(6.5)	3(100)	2(50)	*0.004
Somewhat disagree	13(41.9)	0	1(25)	
Neither agree nor disagree	5(16.1)	0	1(25)	
Somewhat agree	8(25.8)	0	0	
Strongly agree	3(9.7)	0	0	
Comfortable communicating with patients over telehealth				
Strongly disagree	0	2(66.7)	3(75)	*0.001
Somewhat disagree	0	0	0	

Neither agree nor disagree	5(16.1)	1(33.3)	0	
Somewhat agree	11(35.5)	0	1(25)	
Strongly agree	15(48.4)	0	0	
Confidence assessing acute oral and maxillofacial conditions				
Strongly disagree	4(12.9)	2(50)	1(33.3)	*0.024
Somewhat disagree	8(25.8)	2(50)	0	
Neither agree nor disagree	2(6.5)	0	2(66.7)	
Somewhat agree	14(45.2)	0	0	
Strongly agree	3(9.7)	0	0	
Confidence assessing chronic oral and maxillofacial conditions				
Strongly disagree	0	0	0	*0.009
Somewhat disagree	6(19.4)	1(33.3)	3(75)	
Neither agree nor disagree	1(3.2)	2(66.7)	1(25)	
Somewhat agree	20(64.5)	0	0	
Strongly agree	4(12.9)	0	0	

The test applied: Fisher's exact tests, $*P < 0.05$ considered statistically significant *N*
